# Authentic Leadership and Taking Charge Behavior: A Moderated Mediation Model of Psychological Capital and Occupational Calling

**DOI:** 10.3390/ijerph19095492

**Published:** 2022-05-01

**Authors:** Haoluan Fu, Yuechao Du, Zhongming Wang

**Affiliations:** 1 Department of Psychology and Behavioral Sciences, Zhejiang University, Hangzhou 310007, China; fuhaoluan@126.com; 2 Global Entrepreneurship Research Center, Zhejiang University, Hangzhou 310007, China; zmwang_zju@hotmail.com; 3 School of Management, Zhejiang University, Hangzhou 310058, China

**Keywords:** authentic leadership, psychological capital, taking charge behavior, occupational calling

## Abstract

To achieve sustainable development goals, it is necessary to establish a positive organization so that employees can pay attention to their strengths and talents and engage in more proactive behaviors, such as taking charge behavior. Taking charge behavior involves the voluntary and constructive effort of employees to make organizationally functional change, which may consume more scarce resources of employees. Previous studies have shown that support from leaders can promote employees’ taking charge behavior, but most of them are from the perspective of social exchange. By drawing on the conservation of resources theory, we develop a theoretical model in which authentic leadership can provide employees with more positive resources and guide them into gain spiral of resources. We conducted two-wave questionnaire surveys to collect data from 199 employees and their supervisors at 16 companies in China. The results showed that authentic leadership was positively associated with employee taking charge via the mediation role of psychological capital. Furthermore, the direct and indirect relationship between authentic leadership and employee taking charge was demonstrated to be stronger when employees have a higher stage of occupational calling. This study provides a new explanation for the mechanism of authentic leadership and clarifies the boundary conditions of authentic leadership effectiveness.

## 1. Introduction

In the past several decades, topics on sustainable development, such as environmental protection and corporate social responsibility, have received increasing attention from many scholars [1]. The United Nations has proposed 17 sustainable development goals (SDGs), including good health and well-being, decent work and economic growth, and responsible consumption and production [2], which put higher demands on the development model of enterprises, emphasizing the importance of promoting individual, family, and community development at the organizational level [3]. To achieve this goal, we need to build positive organizations that enable employees to continuously focus on their talents and gifts in a positive working environment and organizational culture to achieve high performance, satisfaction, and happiness [4].

Positive organizations provide organizational support to employees, leading them to engage in proactive behavior at work [5], which is referred to as “self-initiated and future-oriented actions that aim to change and improve the situation or oneself” [6]. Such proactive behavior also plays a crucial role in the sustainable transformation of the organization. On the one hand, proactive employees will actively participate in organizational sustainable transformation and hold the assumed responsibility to realize the strategic planning of sustainable transformation [7]. On the other hand, the sustainable development of the organization also requires employees to engage in extra-role behaviors such as voice behavior and organizational citizenship behavior to improve organizational performance and competitive advantage, coping with the constantly changing social environment [8,9]. To further ensure the sustainable transformation of organizations, organizations also need employees to engage in spontaneous and constructive behavior that centers on making change and improvement, which is defined as taking charge behavior [10]. According to Morrison and Phelps [10], employees’ taking charge behavior is different from other proactive behaviors such as organizational citizenship behavior, as it involves the voluntary and constructive effort of employees to go beyond maintaining the status quo, directed at “organizationally functional change” [11,12], which is more consistent with the goal of sustainable transformation of the organization [13]. Therefore, taking charge behavior has recently attracted much attention [14,15,16].

Given the importance of taking charge behavior, researchers have found many antecedents of employees’ taking charge behavior. Cai et al. [17] classified the psychological mechanism that motivates employees to take charge as “can do,” “reason to,” and “energized to.” They found that among the factors at the individual, team, and organizational levels, leadership styles have significant influences on taking charge behavior, especially leadership behaviors such as inclusive leadership [18], empowering leadership [19], and ethical leadership [20]. Although the existing research has illustrated the influence of various types of leadership on taking charge behavior, authentic leadership, as one of the new leadership theories, has received relatively less attention [21]. Authentic leadership is characterized by being true to oneself, integrity, and fairness [22], which plays an important role in influencing individual behavior and the implementation of organizational goals [23]. Several studies have explored the promoting effect of authentic leadership on employees’ taking charge behavior [24,25], but there are some deficiencies. In the selection of a theoretical perspective, researchers have used social exchange theory [26] to explain the promoting effect of authentic leadership on employees’ taking charge behavior, which may not fully reflect the characteristics and essence of authentic leadership. Specifically, the biggest difference between authentic leadership and other types of leadership lies in its sincerity, which is reflected in the transparent relationship, self-awareness, internalized morality, and balanced processing in the interaction with employees, which is difficult to measure simply by cost and reward. In contrast, the role of authentic leadership for employees is more reflected in stimulating their true self, providing security and development space, and enabling employees to have more positive resources to fully realize their self-value [22].

As Luthans and Avolio [27] originally depicted, authentic leadership comes from leaders’ positive psychological resources and in turn leads to the development of themselves and their followers. Therefore, authentic leadership is predicted to result in followers’ positive outcomes by fostering followers’ psychological capacities, which is one of the key outcomes of authentic leadership [28]. Some studies empirically verified this process [29], suggesting that authentic leaders foster followers’ psychological capital by creating a positive organizational climate which is moral, communicative, and supportive [30]. To further integrate and explain the mediating effect of psychological capital, this research will introduce conservation of resource theory (COR). According to COR theory, people are constantly making efforts to preserve, maintain, and construct their valuable resources [31]. However, in a context of constant change, employees’ taking charge behavior will consume more scarce resources of employees, potentially putting them in a loss spiral of resources [32]. To initiate the desired gain spiral of resources, employees will need more support and help from leaders and organizations [33]. Therefore, we assume authentic leadership will play an exemplary role in guiding employees to pay more attention to their own positive psychological resources and cultivate their positive psychological capital; thus promoting their employees’ taking charge behavior.

The interactionist perspective has also been used to view employees’ taking charge behavior, in which not only individual characteristics but also contextual factors influence taking charge behavior [34,35]. Employees’ taking charge behavior often requires not only resources from organizations but also intrinsic motivation from inside [12,36,37]. From the perspective of COR theory, people’s reinvestment of resources depends on their perception of the value and significance of taking charge behavior [18]. Therefore, we assume that employees with high occupational calling, who emphasize realizing self-value through work, will obtain more resources by taking charge at work to enhance the influence of authentic leadership on employees’ taking charge behavior through psychological capital.

This research will contribute to the sustainable development of the organization under the background of sustainable human resource management [38], to further explore the influencing mechanism of how authentic leadership promotes employees’ taking charge behavior based on COR theory. We used two-wave questionnaire surveys to collect data from 199 employees and their supervisors at 16 companies in China. We utilized supervisor–subordinate dyad data to conduct regression analysis. The results not only reveal the positive impact of authentic leadership on employees’ taking charge behavior but also demonstrate the mediating effect of psychological capital and the moderating effect of occupational calling on the relationship between psychological capital and employees’ taking charge behavior. Our study has several theoretical contributions. First, applying COR theory further explains the mechanism by which authentic leadership influences employees’ taking charge behavior and expands the theoretical perspective. Second, it uses psychological capital as a mediating variable and occupational calling as a moderating variable to explore the interaction between authentic leadership and employees’ individual characteristics, which helps us to understand the process mechanism of authentic leadership in a more comprehensive way. Finally, this study provides constructive suggestions on how to promote employees’ taking charge behavior more effectively in the sustainable transformation of enterprises.

## 2. Theoretical Background and Hypothesis Development

### 2.1. Conservation of Resource Theory

The COR theory was originally developed from stress research to better explain individual behavior under stress. The basic assumption of COR theory is that people are always making active efforts to preserve, maintain, protect, and construct what they consider valuable, and these valuable things are resources [31]. The core idea of COR theory is that individuals with more resources are less vulnerable to resource loss and are more capable of investing and gaining additional resources, which forms a gain spiral of resources [39]. Individuals lacking resources, however, are more likely to suffer from pressure caused by resource loss, which leads to insufficient resource investment to prevent resource loss, accelerating resource loss and initiating the loss spiral [40]. 

Positive organizational scholarship calls on research to focus more on positive, rather than negative aspects of stressful situations [41], with an emphasis on health, but not disease, as proposed by the salutogenesis approach [42]. In line with the COR theory, organizations should pay more attention to the generation of resources and to create favorable external conditions for employees to initiate the gain spiral of resources. In that manner, social support brought by leaders can promote the resources available to employees and their appreciation of them, which in turn enhance their sense of self-worth, appreciation, and work meaningfulness [43,44,45]. Employees will also turn such resources into taking charge behaviors, forming a gain spiral of resource acquisition, protection, and construction. In the current study, in line with COR theory, we aim to explain how authentic leadership enhances followers’ psychological capital and activates their taking charge behavior. Psychological capital refers to employees’ positive psychological ability in the working context, and it thus has strong implications for taking charge behaviors. Moreover, we employ employee calling to uncover the boundary condition of the effectiveness of authentic leadership.

### 2.2. Taking Charge Behavior

Taking charge behavior was originally defined as the spontaneous and constructive behavior of organization members, which aimed to change and influence the work behavior of the organization [9]. As an important extra-role behavior, the biggest difference between taking charge behavior and other extra-role behaviors lies in its autonomy, change orientation, and challenge. First, taking charge behavior is discretionary and spontaneous, which is not formally required by others [46]. Second, it is inherently change-oriented and constructive, which means it requires employees to challenge the status quo to take functional change that aims to improve performance [47]. Third, taking charge is more challenging than other extra-role behavior, which requires responsibility and accountability for the possible consequences of the actions [48].

Past research has demonstrated that employees with more self-efficacy and a strong sense of duty are more inclined to take charge [10]. Moon et al. [46] found that, compared to being motivated by personal achievement, duty was positively associated with taking charge. Apart from individual characteristics, contextual factors play an important role in taking charge. For example, leadership such as transformational leadership [19], authentic leadership [25], and inclusive leadership [18], will also promote employees’ taking charge behavior. It appears that taking charge is much more likely to occur when employees believe that the benefits are certain, and the risks are manageable [49]. Based on COR theory, this paper tries to investigate the relationship between authentic leadership and employees’ taking charge from an interactionist perspective, which centers on how employees’ psychological capital is shaped by leaders’ authenticity and thus their reactions.

### 2.3. Authentic Leadership

As stated before, employees’ taking charge behavior often requires organizational support resources and a strong sense of work security [17]. Authentic leadership, defined as “a pattern of leader behavior that draws upon and promotes both positive psychological capacities and a positive ethical climate” [22], has been proven to promote employee proactive behavior [50].

According to Walumbwa’s definition, authentic leadership consists of the following four aspects: self-awareness, relationship transparency, internalized morality, and balanced processing, which all promote employee taking charge behavior.

First, authentic leaders have a strong sense of self-awareness and can objectively understand their various internal characteristics (such as values, strengths, and personality) and the contributions of their subordinates. This gives employees a clearer understanding of their responsibilities and obligations beyond the job requirements. At the same time, the self-awareness of the leader can also promote employees’ self-awareness; thus improving the reasonable cognition of their own abilities in taking charge [51,52].

Second, leaders openly share their inner feelings and thoughts, making the leader–subordinate relationship more transparent. It enables employees to fully obtain feedback from leaders and form a more effective cycle of responsibility, feedback, and incentive, with more recognition of their taking charge behavior. Transparent relationships allow employees to accurately distinguish which behaviors are recognized as proactive behaviors by the leader to avoid being too proactive or interfering with other people’s job responsibilities, which makes employees more determined to take charge voluntarily. Self-awareness and relationship transparency make employees surer of the benefits of taking the initiative, enhance the incentive effect on employees, and enhance their willingness to take the initiative to take responsibility.

Third, authentic leaders tend to maintain maximum objectivity and fairness to the information provided by other members. They are inclusive and open to creative ideas and are willing to adopt even if they are different from their own established beliefs and values. This attitude can create a fair and objective group atmosphere, allowing employees to adhere to their assumed responsibility, which is future-oriented to encompass proactive involvement in future achievement [53].

Finally, internalized morality ensures that leaders are guided by internal ethical standards and values rather than overly influenced by external pressures from peers, organizations, or society. Taking on more responsibilities is often accompanied by taking more risks. Balanced processing and internalized morality can provide employees with a safe working atmosphere and enhance their ability to bear risks [54]; thus improving taking charge. Therefore, we propose the following hypothesis:

**Hypothesis** **1** **(H1).** *Authentic leadership positively influences employee taking charge behavior*.

### 2.4. Psychological Capital as Mediator

Psychological capital was originally defined as a higher-order construct of four psychological capacities: hope, resilience, self-efficacy, and optimism [55]. This construct has been most widely recognized and best fits the positive organizational behavior inclusion criteria [56]. Previous studies have indicated that psychological capital has significant positive effects on employee attitudes, behaviors, and performance [29]. Specifically, employees with high psychological capital are usually characterized as hopeful for targets, confident in abilities, resilient to setbacks, and optimistic to results [57,58].

According to COR theory, when facing work stress, individual characteristic resources can provide support in slowing emotional exhaustion and preventing the generation of stress [31]. Specifically, hopeful people are more confident about the results of resource investment, and they are more willing to invest resources into employees’ taking charge behavior, which is highly expected by organizations. Moreover, self-efficacy also enables employees to make the most of strengths in the process of resource investment; thus improving the resources gained from employees’ taking charge behavior. The individual’s personal values will affect the process of resource evaluation and then affect the individual’s response to stressors [59]. On the one hand, optimistic people will pay more attention to the positive effects brought by employees’ taking charge behavior and less attention to the possible negative effects such as resource loss. On the other hand, resilient people are also better able to withstand the negative impact of resource loss and maintain stable taking charge. Therefore, we derive the following study hypothesis:

**Hypothesis** **2** **(H2).** *Psychological capital is positively related to employees’ taking charge behavior*.

The four capacities of psychological capital are malleable and open to development, but relatively more stable than state concepts such as emotions [55]. For example, resilience can come as much from nurture as from nature [60], as support from leaders and organizations also promotes resilience [61]. Many studies have used psychological capital as the mediator of authentic leadership and employees’ proactive behaviors [29,62]. Most of these studies are based on such a mechanism that authentic leadership comes from leaders’ own positive psychological resources [27], and these leadership behaviors act as models on the psychological resources of employees; thus improving their positive psychological capacities and proactive behaviors [63,64].

In detail, the true self displayed by the leader can make subordinates feel the exemplary role of the leader [65], which not only guides subordinates to focus on their own advantages but also conveys the leader’s confidence in the development of the organization. Paying attention to advantages can also make employees perceive more psychological resources and become more willing to invest resources and initiate the gain spiral. Internalized morality standards also help employees develop a more reliable plan for their goals and remain hopeful in execution [66]. Leaders process information in a balanced way, which will guide employees to focus on the positive factors rather than the negative factors and establish the optimistic attitude of employees. Relationship transparency also enables employees to better understand their leaders’ work arrangements when faced with setbacks and to recover quickly. This will also reduce employees’ excessive concern about resource loss; thus avoiding the loss spiral. Based on this background, we derive the following study hypothesis:

**Hypothesis** **3** **(H3).** *Psychological capital mediates the relationship between authentic leadership and employees’ taking charge behavior*.

### 2.5. Occupational Calling as Moderator

Occupational calling is defined as a transcendent summon experienced as originating beyond the self to approach a particular life role determined in demonstrating or deriving a sense of purpose or meaningfulness, which holds other-oriented values and goals as primary sources of motivation [67]. Occupational calling comes from a certain kind of transcendent guiding force, but it is not only limited to belief or values but also comes from the guidance of social demands to be solved urgently or the internal true self [68]. More importantly, occupational calling contains a strong sense of purpose, which makes individuals having strong altruistic and pro-social tendencies and engage in value-driven behavior when they are engaged in certain occupations [69,70,71]. 

Occupational calling is a concept put forward in the context of Western culture, and the definition itself has a certain religious component. Although Eastern cultures do not have such a religious concept, they do have similar constructs, with connotations closer to a sense of responsibility or duty [72], which was adapted by former research in China [73]. Many studies have explored the joint effects of individual and contextual factors in predicting employees’ taking charge behavior. El Baroudi [74] found that there is an interaction between employees’ perceived occupational calling and mentoring support received from leaders on promoting team member proactivity. Zeng et al. [18] also found that psychological safety and thriving at work both mediate the relationship between inclusive leadership and employees’ taking charge behavior.

From the perspective of COR theory, employees with higher psychological capital have richer psychological resources to invest in employees’ taking charge behavior. However, as McAllister et al. [48] emphasized, challenging behaviors might not be driven in the same way as affiliative behaviors due to the risk inherent in questioning the status quo. We thus posit that the relationship between psychological capital and taking charge might vary depending on contingencies that affect individuals’ perception of the value and significance of taking charge behavior. Individuals with a high degree of occupational calling attach a strong sense of value and meaning to their work, and they pay more attention to the situational information that helps to realize occupational calling in the organization [75]. Therefore, employees with high occupational calling are more likely to feel responsible for improving the performance of the organization [76]. They do not limit their efforts to what is needed to meet organizational requirements and prescribed goals but often go beyond the formal work requirements, making extra efforts for work and wishing to realize their purpose of life through work [77]. Therefore, when they have more psychological resources, they will not hesitate to use them in their work to maximize their self-value through employees’ taking charge behavior. However, employees with a low degree of occupational calling tend to regard work as a way of making a living, and easily satisfied with the status quo. They do not have strong intrinsic motivation to make behaviors beyond the requirements of work. Therefore, even if they receive support and encouragement from the leader, they are easy to choose to ignore and be content. Accordingly, the following hypotheses are proposed in this study:

**Hypothesis** **4** **(H4).** *Occupational calling moderates the relationship between psychological capital and employees’ taking charge behavior*.

**Figure 1:**
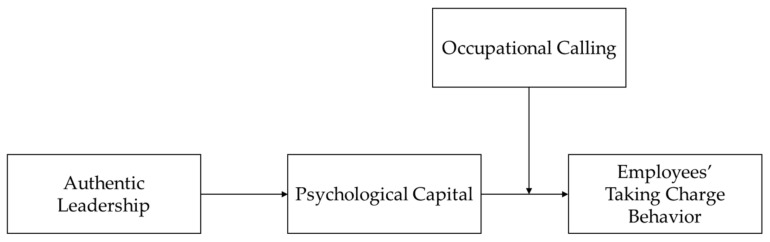
The tested model.

Based on the analysis of the mediating effect and moderating effect, this study proposes a moderated mediation model. Specifically, authentic leaders provide psychological support to employees and improve their psychological resources and psychological capital. However, the occupational calling of employees will affect the way employees deal with social support. Employees with a high degree of occupational calling are more willing to reinvest their psychological resources and create performance and gain returns by taking charge. Psychological capital can provide various resources for them to realize their occupational calling and encourage them to engage in more employees’ taking charge behavior. The higher employees’ occupational calling is, the stronger the indirect influence of authentic leadership on promoting employees’ taking charge behavior through psychological capital, while the lower employees’ calling is, the weaker the indirect influence. Therefore, the following hypotheses are proposed in this study, and Figure 1 shows the proposed model.

**Hypothesis** **5** **(H5).** *Occupational calling moderates the mediating effect of psychological capital on the relationship between authentic leadership and employees’ taking charge behavior such that the mediating effect is stronger when the level of occupational calling is high rather than low*.

## 3. Method

### 3.1. Sample and Procedure

The data were collected from nine companies in software, internet, service, and finance industries in eastern China. To overcome the problem of common method variance as much as possible, we collected data by using the supervisor–subordinate pairing mode [78]. At the beginning, employees and supervisors are informed that all their responses will be used only for academic purposes. Our investigation was conducted in two phases. In the first phase (Time 1), we distributed questionnaires to 310 employees and received 238 valid questionnaires (response rate 78.97%). Employees evaluated their perceptions of authentic leadership, their own psychological capital, occupational calling, and demographic information (gender, age, years of working, and organizational tenure). In the second phase (Time 2), supervisors were asked to evaluate the subordinate’s taking charge behavior. We first wrote the names of specific subordinates on the questionnaire submitted to supervisors and then deleted the relevant information after supervisors completed the questionnaire.

The investigation initially involved 72 supervisors and 310 subordinates. Invalid questionnaires were removed, and 199 matching supervisor–subordinate questionnaires were finally collected from 199 employees and 44 supervisors. The response rate of employees is 83.61% and the response rate of supervisors is 61.11%. On average, each supervisor rated 4.65 employees. Demographically, 55% of respondents are male. The average age of employees is 32.7 years (SD = 7.54). In terms of education, 164 employees had junior college or bachelor’s degrees (82.4%). The average organizational tenure of employees is 2.41 years (SD = 1.95).

### 3.2. Measures

The questionnaires used in this paper are all from previous empirical studies. First, we used a translation–backtranslation procedure to translate the questionnaire [79]. To adapt to the Chinese cultural background, we made appropriate modifications through discussions with experts in related fields. The results showed that only minor changes to the original survey scale were needed. All the participants were asked to respond on a five-point Likert scale, ranging from 1 “strongly disagree” to 5 “strongly agree”, which is different from the 6-point Likert scale as in the Psychological Capital Questionnaire or 7-point Likert scale as in the Calling Scale.

Authentic leadership. Employees evaluated their leaders using a 16-item authentic leadership scale [22]. Example items include “My supervisor seeks feedback to improve interactions with others” and “My supervisor says exactly what he or she means”. Cronbach’s alpha for this measure was 0.88.

Psychological capital. We used a 12-item scale developed by Luthans, Avolio, and Avey [80]. A sample item is “I feel confident in representing my work area in meetings with management”. The Cronbach’s alpha for this measure was 0.94.

Occupational calling. Employees rated their occupational calling using the 12-item scale originally developed by Dobrow and Tosti-Kharas [81]. A sample item is “My career gives me immense personal satisfaction”. The Cronbach’s alpha for this measure was 0.94.

Taking charge behavior. Supervisors were asked to evaluate subordinates’ taking charge behavior based on Morrison and Phelps’s 10-item scale [10]. A sample item is “This person often tries to adopt improved procedures for doing his or her job.” The Cronbach’s alpha for this measure was 0.90.

Control variables. Following previous research [82,83], we controlled for variables that may be related to employee taking charge behavior, including gender, age, education, and organizational tenure.

## 4. Results

### 4.1. Descriptive Statistics

The results of descriptive statistics and correlations of the variables are presented in Table 1. The results preliminarily verified our hypotheses. Specifically, authentic leadership is positively related with psychological capital (*r* = 0.34, *p* < 0.01) and taking charge behavior (*r* = 0.37, *p* < 0.01). Similarly, psychological capital is positively related with taking charge behavior (*r* = 0.34, *p* < 0.01).

We also conducted a multicollinearity test. The results showed that variance inflation factors (VIFs) were all below 10 (1.13 for authentic leadership, 1.13 for psychological capital, and 1.00 for occupational calling), indicating that multicollinearity was not a problem in this study.

### 4.2. Common Method Bias Test

We employed Harman’s single factor test to examine the common method bias. Specifically, all the items of the scales were loaded on factor and the total variance extracted by one factor is only 19.15%, which is far less than 40%, indicating that common method bias is not a serious problem in this study.

### 4.3. Confirmatory Factor Analysis

A confirmatory factor analysis (CFA) was conducted to examine the measurement model fit and the validity of constructs by using Mplus 7.4 (Muthén & Muthén, Los Angeles, CA, USA). As shown in Table 2, the results showed that the four-factor model (authentic leadership, psychological capital, occupational calling, employees’ taking charge behavior) had a much better fit (χ^2^/df = 1.31, CFI = 0.95, TLI = 0.95, RMSEA = 0.04, and SRMR = 0.05) than other models. The results supported that the model we proposed had the best validity.

### 4.4. Hypothesis Testing

Hierarchical regression analysis was conducted by using the SPSS PROCESS macro (IBM, New York, NY, USA) developed by Hayes [84]. Table 3 shows the results of hierarchical regressions. Model 1 regressed the effect of control variables on psychological capital (PC). Model 2 regressed the effect of authentic leadership (AL) and control variables on psychological capital. Model 3 regressed the effect of control variables on employees’ taking charge behavior (TCB). Model 4 regressed the effect of authentic leadership control variables on employees’ taking charge behavior. Model 5 regressed the effect of authentic leadership and psychological capital on employees’ taking charge behavior. Model 6 regressed the effect of authentic leadership, occupational calling (OC) and the interaction (PC×OC) on employees’ taking charge behavior.

The main effect. As shown in Model 4, authentic leadership and control variables are regressed on employees’ taking charge behavior. The result indicates that there is a significant relationship between authentic leadership and taking charge behavior (*b* = 0.41, *p* < 0.01). Thus, hypothesis 1 is supported.

The mediating effect of psychological capital. As shown in Model 5, psychological capital is significantly related to taking charge behavior (*b* = 0.20, *p* < 0.01). Therefore, hypothesis 2 is supported. As shown in Model 2, authentic leadership is significantly related to psychological capital (*b* = 0.43, *p* < 0.01). In Model 5, authentic leadership is also significantly related to taking charge behavior (*b* = 0.32, *p* < 0.01), indicating that psychological capital partially mediates the relationship between authentic leadership and employees’ taking charge behavior [85]. Thus, hypothesis 3 is supported. We adapted the bootstrapping method to further examine the mediating effect [86]. As shown in Table 4, the mediating effect was tested with the expectation that the indirect effect should not be zero [87]. The result shows that the index of the indirect effect of authentic leadership on taking charge behavior via psychological capital is 0.09 (95% CI [0.02, 0.20]).

**Figure 2:**
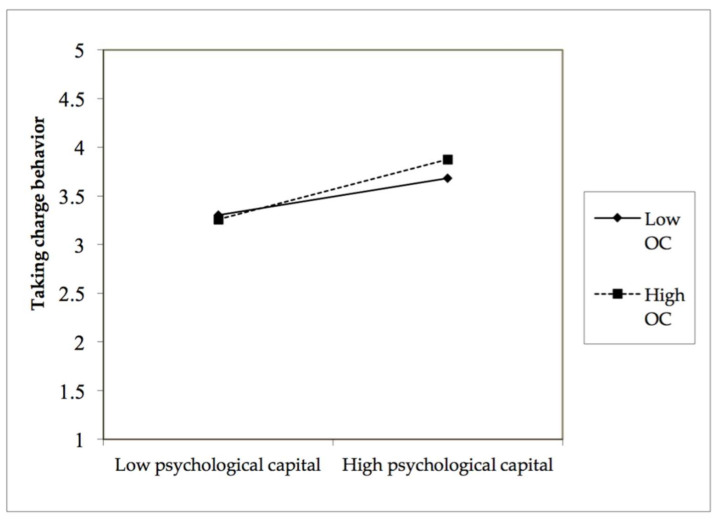
The moderating effect of OC on PC and TCB.

The moderating effect of occupational calling. As shown in Model 6, the interaction between psychological capital and occupational calling is significantly correlated with employees’ taking charge behavior (*b* = 0.13, *p* < 0.01). Thus, hypothesis 4 is supported. To further show the moderating effect of occupational calling, we plotted the effect of psychological capital on taking charge behavior based on a high versus low level of occupational calling. As shown in Figure 2, the plot indicates that psychological capital has a stronger effect on employees’ taking charge behavior when the occupational calling is high rather than low. Furthermore, we also tested the moderating effect of occupational calling on the relationship between authentic leadership and employees’ taking charge behavior via psychological capital. Table 5 shows the conditional indirect effect of authentic leadership on employees’ taking charge behavior for different values of occupational calling (mean minus one SD as Low; mean plus one SD as High). As shown in Table 6, the index of moderated mediation effect for employees’ taking charge behavior is 0.06, with confidence intervals excluding zero, supporting hypothesis 5.

## 5. Discussion

### 5.1. Theoretical Implications

This study expands the theoretical perspective of authentic leadership influencing employees’ taking charge behavior and has several theoretical implications. First, many scholars have explored the influence of authentic leadership on employees’ proactive behaviors, such as voice behavior and organizational citizenship behavior, without paying enough attention to taking charge behavior [50]. Only a few studies have focused on the relationship between authentic leadership and employees’ taking charge behavior [25], but most of them are conducted from the perspective of social exchange theory and social information processing, which cannot fully reflect the characteristics and essence of authentic leadership [88]. This study, based on COR theory, finds that authentic leadership can make employees more convinced of the return on investment brought by taking on more responsibilities and actively taking possible risks to stimulate employees’ taking charge behavior. Such a finding supports the underlying assumption of COR theory that social support brought by leaders can promote the resources available to employees and then help them initiate the gain spiral [34]. The findings provide important theoretical insight into why subordinates of authentic leaders are more inclined to take charge.

Next, psychological capital is used as a mediator to investigate the mechanism of the effect of authentic leadership on employees’ taking charge behavior, answering the call for an integrative approach to authentic leadership and psychological capital research [54]. Although several studies have used psychological capital as a mediator to explore the influence of authentic leadership on proactive behavior [88,89], few studies have used COR theory to explain this mechanism. Psychological capital, as a trait-like variable, can be developed and applied when employees possess a certain degree of positive psychological resources. The leader’s authentic behavior can provide more psychological resources for employees, and their true self plays an exemplary role, encouraging employees to explore and make use of their own advantages. This study explains the effect of authentic leadership on employees’ taking charge behavior from the perspective of COR theory, enriching the integrative approach to authentic leadership and psychological capital.

Finally, the examination of the moderating effect of employee occupational calling in this study responds to the call of previous scholars to further explore the boundary conditions of the effect of authentic leadership on employees’ taking charge behavior. Incorporating employee occupational calling into the research framework and considering the interaction between authentic leadership and employees’ individual characteristics is more conducive to integrating the influence of organizational factors and individual factors from an interactive perspective. The results are helpful for understanding the effect and mechanism of authentic leadership in a more comprehensive way. Moreover, this study focuses on the reinvestment process after resource acquisition in COR theory. It is found that when employees highly recognize the sense of meaning and value in work (feeling calling), they are more willing to reinvest resources in work and taking charge. This provides more empirical support for the hypothesis of COR theory on the process of resource reinvestment. In addition, most studies have focused on the antecedents and influencing factors of calling [90] and paid less attention to the regulatory role of calling. This paper extends calling to the field of authentic leadership and employees’ taking charge behavior and enriches the empirical research of calling.

### 5.2. Practical Implications

Employees’ taking charge behavior plays an important role in the sustainable development of organizations and the enhancement of organizational cohesion. Our study provides management implications for organizations on how to promote employees’ taking charge behavior. First, organizations should pay attention to the authentic leadership development of leaders at different levels, improve the positive psychological resources of leaders themselves, and then encourage leaders to engage in authentic leadership behavior. This study found that authentic leadership has a positive effect on employees’ taking charge behavior, so organizations should establish an effective personnel selection and talent management system to promote leaders with authentic leadership. On the other hand, training programs should pay attention to the cultivation of authentic leadership and guide them to influence employees through their real selves to build a more transparent and high-quality relationship between leaders and subordinates. At the same time, authentic leadership itself can also provide human resource advantages for the long-term sustainable development of enterprises.

Second, we should strengthen the development of the psychological capital of employees. The results show that psychological capital mediates the promotion effect of authentic leadership on employees’ taking charge behavior, so managers should pay attention to the change in employees’ psychological capital in the process of showing their true self and achieving authentic leadership behavior. In the process of interaction between leaders and employees, they need to encourage employees to develop their own positive psychological capacities and promote the full use of their psychological resources. At the same time, in sustainable human resource management activities, organizations should constantly improve employees’ sense of efficacy and sense of hope for goals, help employees deal with difficulties in a resilient way, pay more attention to positive factors rather than negative ones, and thus continue to develop psychological capital for employees and promote employees’ taking charge behavior.

Third, attention should be given to the occupational calling of employees. Employees with high occupational calling are more likely to be positively influenced by authentic leadership. Therefore, more attention should be given to the level of employees’ occupational calling and a working environment conducive to their pursuit of work value. In terms of enterprise recruitment and promotion, organizations should not only examine the professional knowledge and skills of employees but also attach importance to measuring their occupational calling, promote employees who can realize their calling at work, and create a work atmosphere with mission and vision.

### 5.3. Limitations and Future Directions

This study has the following limitations and deficiencies. Based on COR theory, this study explores the mediating role of psychological capital between authentic leadership and employees’ taking charge behavior. All the variables and theories are proposed under Western culture, while the subjects are from China. This may not generate universal conclusions. Future research may validate this model in different culture backgrounds, to obtain more general conclusions. There may be other theoretical perspectives and mechanisms for the influence of authentic leadership on employees’ taking charge behavior. Combined with Chinese cultural characteristics, future studies can take power distance, collectivism, guanxi, traditionalism, and other variables to obtain more illuminating conclusions.

This study examined the moderating effect of employees’ personal characteristics on the effect of authentic leadership. According to COR theory, employees constantly acquire resources at work and create more resources in the investment process. Other individual factors in addition to occupational calling may also affect employees’ resource acquisition and investment processes, leading to the gain spiral of resources. On the other hand, there may be some personal traits that make employees more likely to initiate loss spirals and hinder employees from receiving support from the organization. Future research can further explore the interaction between organizational and individual factors, expanding the depth and breadth of this mechanism.

The present study analyzed the effects of authentic leadership on employees’ taking charge behavior at the micro level. However, as Bandura [91] suggested, social phenomena often occur in groups, and these group interactions influence the very nature of psychological constructs. Authentic leadership and psychological capital are both conceptualized as being multilevel [92], and future research may explore their relationship at the group level, combining with concepts such as organizational resilience to investigate from the perspective of the functioning of the organization. Moreover, although this study adopts a longitudinal study of two phases, all the measurement methods used in this study are questionnaires, only reflecting the subjective feelings of the participants In future research, more diversified measurement methods, such as implicit psychological capital tests and situational judgment tests, can be used.

## 6. Conclusions

Based on COR theory, this paper explored the effect of authentic leadership on employees’ taking charge behavior through psychological capital and examined the moderating effect of occupational calling. First, the empirical research results show that authentic leadership can positively influence employees’ taking charge behavior because authentic leadership can provide employees with strong organizational resources and create a safe working atmosphere so that employees will actively take more responsibilities at work and take the initiative to engage in employees’ taking charge behavior. Therefore, providing employees with such a supporting working environment and organizational culture is essential in building positive organizations.

Second, psychological capital acts as a mediator in the relationship between authentic leadership and employees’ taking charge behavior. In addition to directly promoting employees’ taking charge behavior, authentic leadership can also influence employees’ taking charge behavior indirectly through psychological capital. Authentic leadership is derived from the positive psychological resources of leaders and acts on the positive psychological resources of employees, so it can help employees better develop their positive psychological capacities. Positive psychological capacity is a necessary precondition for employees to have sufficient resources for reinvestment and thus to engage in more taking charge behavior.

Third, the higher the occupational calling of employees is, the stronger the effect of authentic leadership on employees’ taking charge behavior will be through psychological capital. Individuals with high occupational calling will respond more to the support from authentic leadership and recognize the authentic behavior of leaders, which is beneficial for employees to make full use of positive psychological resources and reinvest in subsequent employees’ taking charge behavior. By introducing COR theory, this study provides a new theoretical perspective for promoting the flourishing development of employees and emphasizes the important role of the exploration and exploitation of psychological resources.

## Figures and Tables

**Table 1:** Descriptive statistics and intercorrelations of variables.

Variable	Mean	SD	1	2	3	4	5	6	7
1. Gender	0.45	0.50							
2. Age	32.77	7.54	0.02						
3. Edu	3.50	1.03	0.03	0.10					
4. OT	2.41	1.95	0.02	0.27 **	−0.03				
5. AL	4.20	0.61	−0.04	−0.01	0.02	−0.03			
6. PC	4.08	0.77	−0.11	−0.08	0.01	−0.03	0.34 **		
7. OC	3.98	0.94	−0.12	0.07	−0.04	−0.03	0.01	0.04	
8. TCB	4.16	0.69	−0.11	−0.07	−0.07	−0.06	0.37 **	0.34 **	−0.09

*N* = 199. OT represents organizational tenure; Edu represents education; AL represents authentic leadership; PC represents psychological capital; OC represents occupational calling; TCB represents taking charge behavior. ** *p* < 0.01.

**Table 2:** Confirmatory factor analysis.

Factor Structure	χ^2^/df	CFI	TLI	RMSEA	SRMR
Four-factor model (AL; PC; OC; TCB)	1.31	0.95	0.95	0.04	0.05
Three-factor model (combining AL and OC together)	2.86	0.71	0.58	0.10	0.15
Three-factor model (combining AL and PC together)	2.25	0.81	0.63	0.08	0.08
Three-factor model (combining OC and PC together)	2.85	0.72	0.58	0.10	0.15
Two-factor model (combining AL, PC, OC together)	3.79	0.57	0.48	0.12	0.17
One-factor model (combining all items into one factor)	4.76	0.42	0.39	0.14	0.18

Note. AL represents authentic leadership; PC represents psychological capital; OC represents occupational calling; TCB represents taking charge behavior. CFI, Comparative Fit Index; TLI, Tucker–Lewis Index; RMSEA, root mean squared error of approximation; SRMR, standardized root mean square residual.

**Table 3:** Regression results of the mixed model.

	Psychological Capital			Taking Charge Behavior		
	M1	M2	M3	M4	M5	M6
CV						
Gender	−0.16	−0.14	−0.15	−0.13	−0.10	−0.13
Age	−0.01	−0.01	−0.01	−0.01	−0.01	−0.01
Edu	−0.02	0.01	−0.04	−0.05	−0.05	−0.05
OT	−0.01	0.01	−0.01	0.01	−0.01	−0.02
IV						
AL		0.43 **		0.41 **	0.32 **	0.33 **
Mediator						
PC					0.20 **	0.21 **
Moderator						
OC						0.08
Interaction						
PC×OC						0.13 **
R^2^	0.02	0.13	0.02	0.15	0.20	0.23
F	0.70	4.92 **	0.94	5.81 **	6.82 **	7.44 **

Note. *N* = 199. CV represents control variable; IV represents independent variable; OT represents organizational tenure; Edu represents education; AL represents authentic leadership; PC represents psychological capital; OC represents occupational calling. ** *p* < 0.01.

**Table 4:** Regression analysis of the mediating effect.

Effect	B	SE	LLCI	ULCI
Direct effect of X on Y	0.32 **	0.07	0.17	0.48
Indirect effect of X on Y	0.09 **	0.04	0.02	0.20
Total effect of X on Y	0.41 **	0.08	0.16	0.65

Note. ** *p* < 0.01.

**Table 5:** Conditional indirect effect at specific values of occupational calling.

Moderator	Effect	SE	LLCI	ULCI
Low	0.04	0.04	−0.02	0.13
Mean	0.09	0.04	0.03	0.21
High	0.14	0.07	0.04	0.31

**Table 6:** Index of moderated mediation.

Outcome	Index	SE	LLCI	ULCI
TCB	0.06	0.04	0.01	0.15

## Data Availability

The data presented in this study are available on request from the corresponding author. The data are not publicly available due to respondents’ privacy.
